# Mother Vocal Recognition in Antarctic Fur Seal *Arctocephalus gazella* Pups: A Two-Step Process

**DOI:** 10.1371/journal.pone.0134513

**Published:** 2015-09-02

**Authors:** Thierry Aubin, Pierre Jouventin, Isabelle Charrier

**Affiliations:** 1 Université Paris Sud, Neuro-PSI, UMR 9197, Orsay, F-91405, France; 2 CNRS, Orsay, F-91405, France; 3 CEFE, UMR 5175, Montpellier, F-34293, France; Sonoma State University, UNITED STATES

## Abstract

In otariids, mother’s recognition by pups is essential to their survival since females nurse exclusively their own young and can be very aggressive towards non-kin. Antarctic fur seal, *Arctocephalus gazella*, come ashore to breed and form dense colonies. During the 4-month lactation period, females alternate foraging trips at sea with suckling period ashore. On each return to the colony, females and pups first use vocalizations to find each other among several hundred conspecifics and olfaction is used as a final check. Such vocal identification has to be highly efficient. In this present study, we investigated the components of the individual vocal signature used by pups to identify their mothers by performing playback experiments on pups with synthetic signals. We thus tested the efficiency of this individual vocal signature by performing propagation tests and by testing pups at different playback distances. Pups use both amplitude and frequency modulations to identify their mother’s voice, as well as the energy spectrum. Propagation tests showed that frequency modulations propagated reliably up to 64m, whereas amplitude modulations and spectral content greatly were highly degraded for distances over 8m. Playback on pups at different distances suggested that the individual identification is a two-step process: at long range, pups identified first the frequency modulation pattern of their mother’s calls, and other components of the vocal signature at closer range. The individual vocal recognition system developed by Antarctic fur seals is well adapted to face the main constraint of finding kin in a crowd.

## Introduction

Otariids are social animals that use vocal signals in several social contexts such as territorial and female defense, aggressive behavior and mother-pup identification (for a review see [1]). Most studies on otariids have focused on mother-pup recognition since, as other social and colonial species, they have developed strong abilities to identify each other [2]. Individual recognition induces mutual benefits[3]: females limit their energy expenditure by avoiding misdirected maternal care, and pups decrease the risk of injury by approaching non-mother females. In otariids, recognition of mothers by pups is essential to pup survival as females exclusively nurse their own young [4] and can be very aggressive towards non-kin [5,6].This is exacerbated by the repeated and frequent maternal absences during the course of lactation: otariid mothers alternate foraging trips to sea (1–20 days depending on species) with bouts of suckling ashore (1–5 days). For these reasons, an efficient individual recognition process is needed in order for a pup to safely relocate its mother in the crowded colony. Individual recognition can rely on different sensorial modalities: audition, olfaction, and vision. However the acoustic channel appears to be the most reliable one in a colonial environment. Indeed, pinnipeds develop myopia and astigmatism in air [7] and olfactory cues are efficient only at short distance (within 15 cm), when mother and pup are in contact [8–11]. While olfactory and visual cues are efficient at short distances, acoustic signals can be well transmitted over longer distances (from 32 to 128m [12–14]). Vocal recognition between mother and young has been observed and experimentally demonstrated in several pinnipeds species (see [1] for review, [12–15]), however, the fine acoustic mechanisms involved in individual vocal identification are only known in two species, the Subantarctic fur seal, *Arctocephalus tropicalis* [16,17] and the Australian sea lion, *Neophoca cinerea* [12,18].

Antarctic fur seals, *Arctocephalus gazella*, breed on land and form dense colonies. A few days after their arrival in the colony, females give birth to a single pup that they will suckle for 4 months [19]. During the entire lactation period, females alternate foraging trips (4–7 days) with periods ashore suckling (1–3 days) [19–21]. On each return to the colony, females and pups have to find each other among several hundred conspecifics; this identification being mainly mediated by vocal signals [22,23]. Considering the strong social and environmental constraints found in this species (high density of animals, no fostering, noisy environment), the individual vocal signature must be highly individualized and also resistant to degradation during propagation. In the present study, we investigate the process of recognition of mothers’ voices by pups. By performing playback experiments on pups using synthetic signals, we first demonstrate the acoustic features involved in the vocal identification of mothers. Second, we tested the efficiency of this individual vocal signature by analyzing propagated signals to characterize the call’s degradations undergone during propagation throughout the colony, and by performing playback tests at different emitter-receiver distances.

## Materials and Methods

### Subjects and Study Area

Recordings and experiments were carried out on Kerguelen Archipelago at Cape Noir in the Courbet Peninsula (49°4.2’S latitude, 70°27.3’E longitude) in January and February 2000 during the period of lactation. The studied colony was composed of 750 pairs of mothers and pups. Tested pups were between 3 and 6 weeks old. Pups were tagged on both fore flippers by using individually numbered plastic tag (Dalton Rototags, Dalton Supply, Nettlebed, UK) and mothers were marked by a small colored spot painted on the back. Permission to conduct the study on Kerguelen Archipelago was given by IPEV (program ETHOTAAF n°354) and Terres Australes et Antarctiques Françaises (TAAF). Animal handling and acoustic experiments carried out in this study complied with current French laws and were approved by the Ethical Committee of the French Polar Institute (IPEV). Our study did not involve endangered or protected species, and all sampling procedures (sound recording) and experimental procedures (playback) were specifically approved as part of the field permit.

### Recording and Playback Equipment

Pup attraction calls produced by 32 females when searching for their own pup were recorded with a Sony TCD10 Pro II DAT (frequency response flat within the range 20–20.000Hz) and an omnidirectional Beyer M 69 microphone (frequency response 100–20.000 Hz, at ±1 dB). The microphone was mounted on a 3m pole, so that animals could be approached without disturbance, and the distance between the recorded female and the microphone was approximately 1m.

Signals were broadcast with the DAT connected to an Audax unidirectional loudspeaker via a customized 20-W amplifier (frequency response 100–5.600 Hz ± 2 dB). During propagation tests, natural signals were broadcast and re-recorded at different distances from the loudspeaker by using the aforementioned omnidirectional Beyer M 69 microphone connected to a second Sony TCD10 Pro II DAT. For sound pressure level measurements (SPL in dB) we used a 4176 Brüel and Kjaer Sound Level Meter (linear scale, slow settings).

### Acquisition and Signal Synthesis

Analog signals were digitized at a 22.050 Hz sampling rate. Pup attraction calls produced by females to relocate their pups are complex signals composed of a fundamental frequency and its relative series of harmonics modulated in amplitude and frequency.

**Fig 1:**
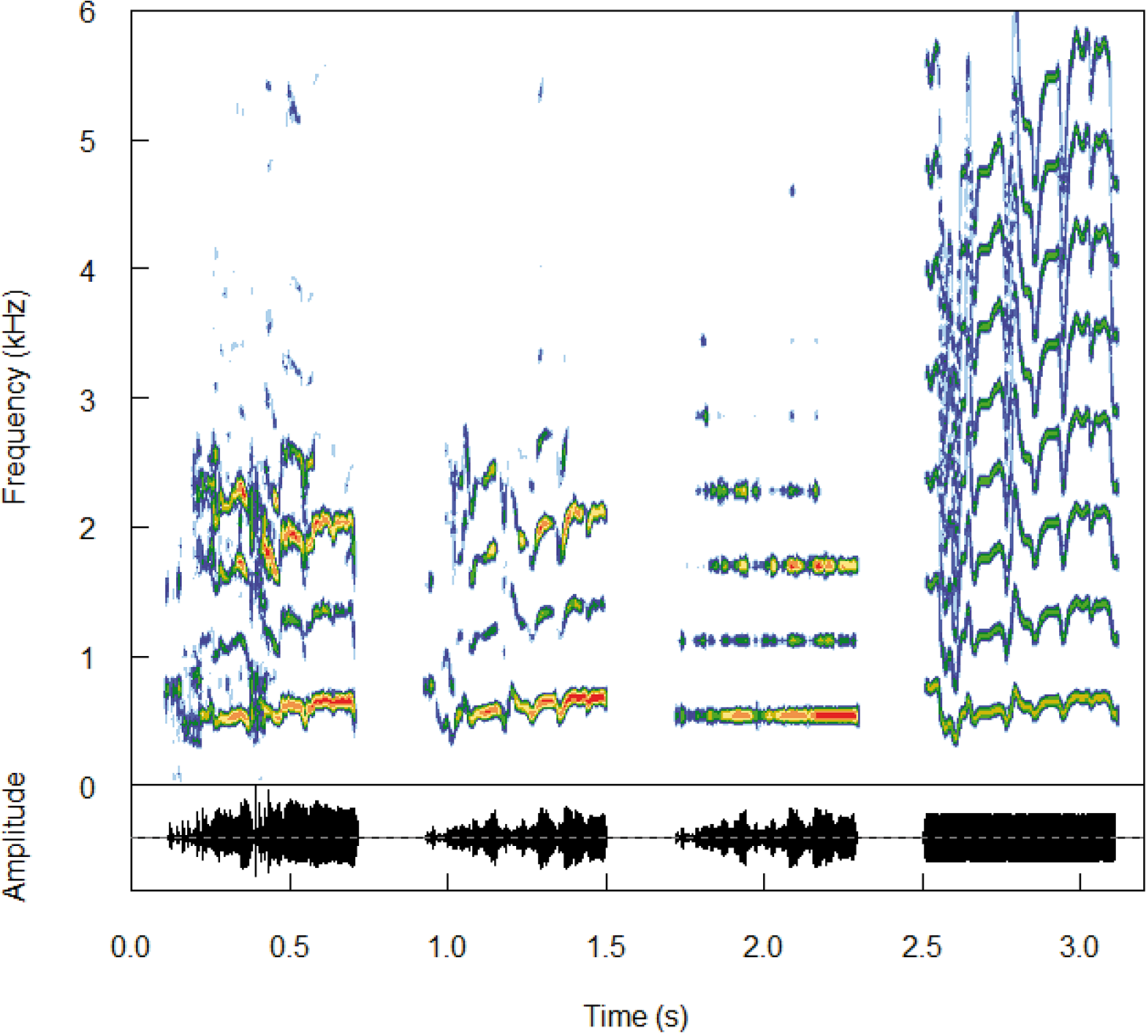
Experimental signals modified in the temporal domain. From left to right: natural control, synthetic control, wfm (no frequency modulation), and wam (no amplitude modulation).

*The synthetic control* is a female’s call entirely synthesized on the basis of a natural call (Fig 1). Synthetized calls were built using the Avisoft graphic synthesizer that extracts the frequency contour of the fundamental frequency and the relative amplitude of the different harmonics of the calls. This extracted information was thus used to synthesize the synthetic controls. Spectrogram correlations between the natural and synthetic controls were performed to control their similarity using the Avisoft correlator on a sample of 14 female calls (mean ± SE = 0.857 ± 0.098). For each female, we built 3 different versions for the synthetic control based on 3 different natural calls (*synt1*, *synt2 and synt3*). This was done to increase the diversity of tested signals avoiding habituation and pseudo-replication effect. Pups were tested with the different synthetic controls on which experimental signals were based on. Since all the synthetic controls elicited responses similar to those obtained with natural calls (see results section), we built 10 different experimental signals based on these synthetic controls.

### Experimental Signals

To assess the temporal features such as the frequency and the amplitude modulation involved in the vocal identification of mothers by pups, we built three experimental signals (Fig 1). One experimental signal was built by attributing the same constant amplitude to each harmonic but by keeping its natural frequency modulation (*wam*). A second type of signal was built by removing the frequency modulation but by keeping the natural envelope (*wfm*). The carrier frequency was a harmonic series and the value of the fundamental corresponded to the mean value of the fundamental frequency of the mother call. We applied to this carrier frequency the natural envelope that was extracted from the mother call, using the Hilbert transform calculation [24,25]. Finally, a third signal was built by time-reversing the signal and thus modifying both frequency and amplitude modulation patterns (*rev*).

**Fig 2:**
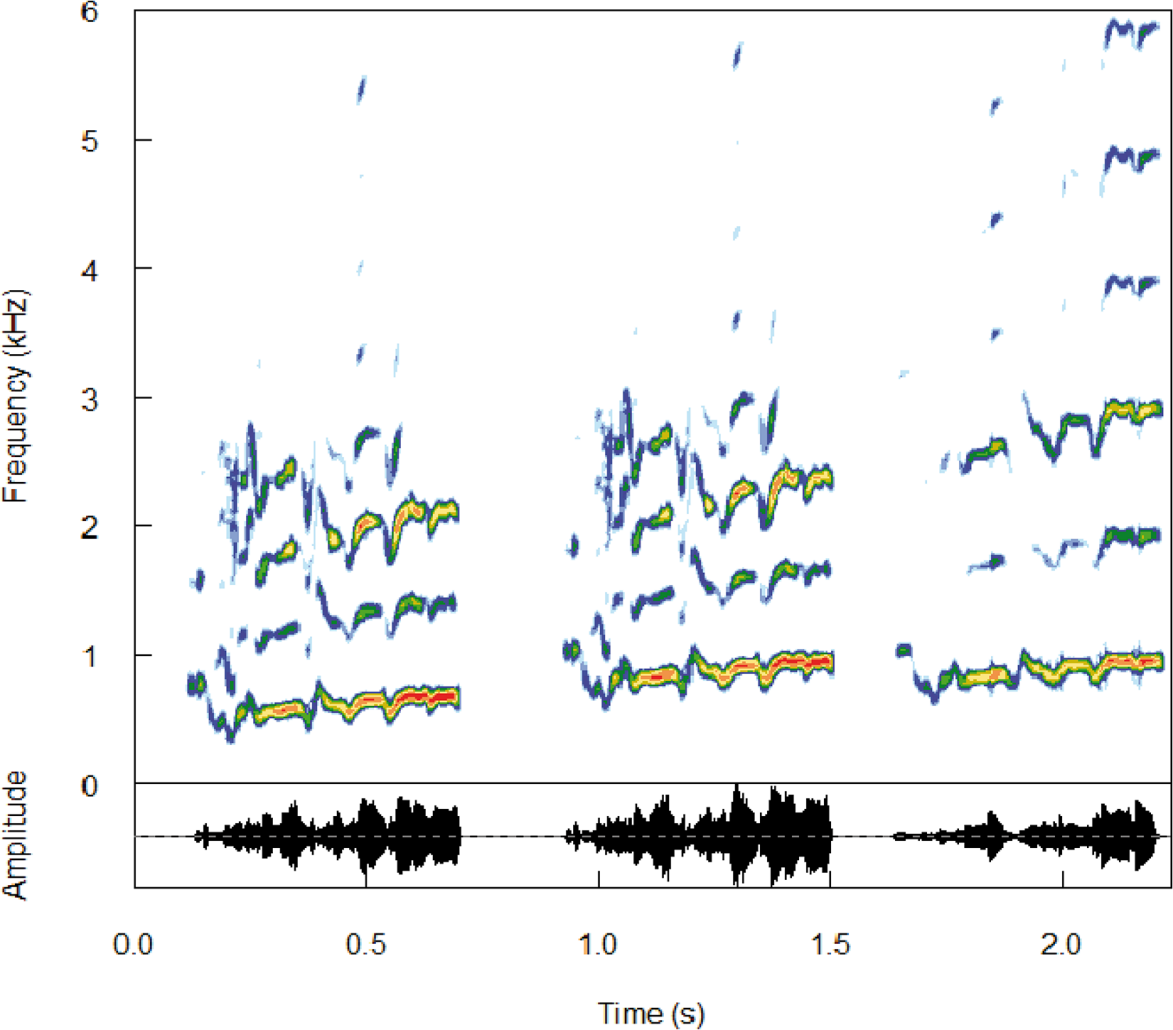
Experimental signals modified in the frequency domain. From left to right: synthetic control, l300 (linear shift: entire frequency spectrum shifted up by 300 Hz), a300 (additional shift: fundamental frequency shifted up by 300 Hz).

Similarly, we investigated the frequency features of the calls involved in the vocal recognition of mothers by pups, and we built seven experimental signals in which a given parameter was modified (Fig 2). Three main types of signals were built. One type consisted of linearly shifting the entire synthetic control upwards by 75, 150 and 300 Hz (linear shifts: *l75*, *l150 and l300*). Linear shifts were done by picking a data record through a square window, applying short-term overlapping (50%) Fast Fourier Transform (FFT), followed by a linear shift of each spectrum, and by a short-term inverse Fast Fourier Transform (FFT-1, [26]). Such signals allowed us to determine if pups performed an accurate frequency analysis of their mother calls. A second type consisted of linearly shifting the fundamental frequency upwards by 75, 150 and 300 Hz (additional shifts: *a75*, *a150 and a300*), but the series of relative harmonics was rebuilt in order to keep the relationship between the fundamental and its harmonics (e.g., if the original fo value was 500 Hz, we shifted it upwards by 75 Hz, fo’ = 575 Hz then the next harmonics values were 1150, 1725, 2300, 2875 Hz…). By using such signals, we can assess if pups pay attention to the frequency difference among harmonics to identify their mothers. A third type of signal in which all frequency values were kept from the natural signal but the amplitude level of each harmonic was randomly changed (*sort*). With Avisoft synthesizer, we were able to save the relative amplitude pattern of each harmonic, and thus we could randomly attribute the amplitude pattern of a given harmonic to another one. By doing this, the overall envelope of the call did not change. This was done to test the relative importance of the energy spectrum in the individual recognition process.

Experimental signals *wam* and *wfm* were built from *synt1*; *rev*, *l75* and *a75* from *synt2*; *l150*, *l300*, *a150* and *a300* from *synt3*; and *sort* either from *synt2* or *synt3*.

Experimental signals *synt1*, *synt2*, *synt3*, *wam*, *a75*, *a150*, *a300* and *sort* were built using the graphic synthesizer facility of Avisoft SAS Lab Pro (Avisoft Bioacoustics, R. Specht–version 3.74) and *wfm*, *rev*, *l75*, *l150 and l300* using Syntana [27].

### Playback Procedure

Pups were tested in the absence of their mother when they are foraging at sea (4–7 days). As a general rule, for a given pup and for a given playback session, we broadcast an experimental tape containing 2 to 5 experimental series (one series: one modified synthesized signals repeated 10 times, each signal being separated by 4s, mean duration: 50 s) and one control series (one synthesized call from the mother repeated 10 times, each call being separated by 4s, series duration: 50 s). Within a playback session, there was always a pause of at least 5 minutes between each playback series. The series presentation order was randomized between playback sessions. To avoid habituation of pups, there was always a minimum of 2 days between playback sessions and a given pup was only tested once with a given experimental series. We tested 9 to 16 pups for each experimental series, each focal pup being tested with 5 different experimental series on average (range: 1–8) over several sessions. Signals were played back at a natural SPL (84 ± 4 dB at 1 m), and the loudspeaker was placed 7 m from the tested pup. A total of 29 pups were used in this experiment.

### Behavioural Responses to Playback Tests

The behavioral responses of pups to playbacks were characterized by 3 measures: number of emitted calls (NC), latency to call (LC) and latency to look (LL) at the loudspeaker. Instead of separately analyzing these 3 behavioral measures, we collapsed them using a principal component analysis (PCA, varimax rotation). The PC scores of principal components showing eigenvalues > 1 were then compared using a Wilcoxon matched-pairs test (two-tailed test) to determine whether behavioral responses differed between synthetic controls and experimental signals. We used Holm's Sequential Bonferroni Procedure to adjust p values for multiple comparisons.

### Signal Propagation

#### Analyses of degraded signals

In the second experiment, we played back one series composed of a representative natural female call (i.e. at the level of amplitude and frequency modulation patterns, see Fig 1) repeated 20 times at 5 different propagation distances: 1 (control), 8, 32, 64 and 128 meters, and re-recorded these series to analyze the propagated calls. Calls were propagated beside the colony, on a flat meadow showing the same characteristics than the one used by mother-pup pairs during the lactation period. For each distance, only the 12 propagated calls with the best signal/noise ratio were kept for the analyses. For each distance, the recorded calls were then examined in the frequency versus amplitude domain on the averaged spectrum of the 12 calls, in the time versus amplitude domain on their averaged envelope and in the time versus frequency domain on their averaged FM pattern (measured on spectrograms). We compared the averaged parameters at a given propagation distance to those obtained at 1 m (control) by using Pearson’s product-moment correlation coefficient.

#### Tests on pups

When waiting for their mothers, pups are often gathered in small groups (5 to 12 individuals on average in the studied area). To assess the responsiveness and selectiveness of pups towards their mother’s calls, we performed one additional playback experiments. Pup attraction call series from 12 different females (one series consisted of one calls repeated 10 times as previously used in other playback experiments) were played back at different distances (8, 32 and 64 meters) to 12 different groups of pups of similar size (about 10 individuals in each group including the pup whose mother’s calls were used). We counted the number of pups responding to a given series of calls at the 3 different distances. During each playback session, only one series was broadcast, and we used the calls of a mother whose pup was in the tested group. This experiment allowed us to determine the discrimination abilities of pups in regards to distances, and thus the rate of recognition error by pups with distances. Indeed, we expected several pups responding at long distances whereas only one should respond at the shortest distance. The results of this experiment should reflect the efficiency of the individual vocal signature to environmental constraints. To compare the number of pups responding to natural females’ calls at the 3 broadcast distances, we performed a Friedman ANOVA. All statistical analyses were performed using Statistica (Statsoft Inc., version 6.0).

## Results

### Experimental Signals

Only the first component of the PCA performed on the 3 behavioural measurements showed an eigenvalue greater than 1 and explained 73.9% of the total variance. All were strongly correlated to PC1, with LC and LL negatively correlated to PC1 (-0.85 and -0.82 respectively, 0.91 for NC). Positive PC scores indicate a strong reaction, with shorter latencies to call and to look towards the speaker, and more calls in response. Comparisons of pups’ behavioural responses obtained with the controls and the different experimental signals are summarised in Table 1.

**Table 1:** Behavioural responses obtained with natural, synthetic control and experimental series on pups. Comparison of behavioural scores obtained between 1) the synthetic and the natural controls, and 2) the synthetic controls and the modified signals (Wilcoxon matched pair tests, p values were adjusted using the Holm's Sequential Bonferroni Procedure, T and Z are statistic values for n<15 and n>15 respectively). Behavioural scores obtained with modified signals were compared to the average scores obtained with the different synthetic signals (Moy. Synth).

	N	T	Z	Adjusted P values
**1- Synthetic Signals**				
Natural Control vs Synt1	14	28	0.444554	0.656642
Natural Control vs Synt2	14	27	1.600800	0.109422
Natural Control vs Synt3	13	28	1.222999	0.221331
Natural Control vs Mean Synth	27	148	0.985025	0.324613
**2- Modified Signals**				
**Time Domain**				
wam	16	0	3.516196	**0.000438**
wfm	16	11	2.947400	**0.003205**
rev	16	7	3.154235	**0.001609**
**Frequency Domain**				
l75	10	13	1.477977	0.139415
l150	11	2	2.756236	**0.005847**
l300	9	1	2.547100	**0.010863**
a75	10	17	1.070259	0.853512
a150	10	5	2.293412	0.065475
a300	9	1	2.547100	**0.010863**
sort	13	1	3.109912	**0.001872**

### Natural Control Versus Synthetic Controls

**Fig 3:**
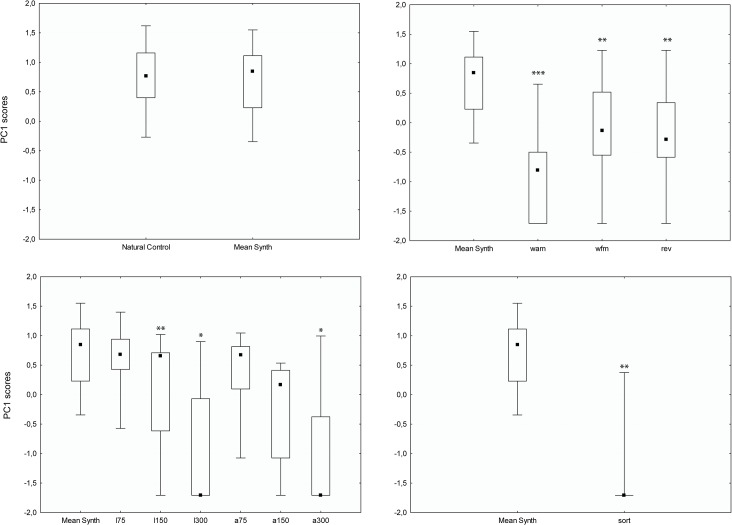
Behavioural scores (PC1) obtained with the natural controls, synthetic controls and the different experimental signals. Abbreviations: wam (no amplitude modulation); wfm (no frequency modulation); rev (time-reversed signal); l75,l175, 300 (linear shifts: entire frequency spectrum shifted up by 75, 150 and 300 Hz respectively); a75, a150, a300 (additional shift: fundamental frequency shifted up by 75, 150 and 300 Hz respectively). Boxplots indicate medians (square markers) and 25–75 percentiles, and whiskers show min-max values of PC scores. P-values resulting from the comparison of responses between the synthetic controls (Mean Synth) and the different experimental signals are indicated as * for p < 0.05, ** for p < 0.01 and *** for p < 0.001.

Responses elicited with the different synthetic controls were similar to those obtained with the natural controls (Wilcoxon matched pairs tests, p values ranging from 0.109 to 0.656, Table 1 and Fig 3). These tests allowed us to validate our synthetic calls, pups showing no significant differences in response to natural or synthetic mother’s calls. Considering this, we calculated an overall score of the different responses obtained with the different synthetic signals for each pup (i.e., pups were tested with 1, 2 or 3 synthetic signals), and we used such average scores (mean Synth.) for comparison to those obtained with modified signals.

### Signals Modified in the Time Domain

Responses triggered with both signals without amplitude modulation (*wam*) or without frequency modulation (*wfm*) were significantly different from those obtained with synthetic controls (Wilcoxon matched pair tests, p values < 0.005–Table 1 and Fig 3). A similar result was found with the time-reversed calls (*rev*, p = 0.0016, Table 1 and Fig 3).

### Signals Modified in the Frequency Domain

Only the linear shift *l75* elicited strong responses (Wilcoxon matched pair tests, p = 0.28 and Fig 3), whereas responses to both *l150* and *l300* were found significantly different from those obtained with the synthetic controls (p values < 0.01, see Table 1 and Fig 3). With the other set of shifted signals (*i*.*e*., *additional shifts*), both *a75* and a 150 still triggered recognition (p values > 0.50, see Table 1 and Fig 3) whereas responses to *a300* signals were found significantly different from the synthetic controls (p = 0.01, Table 1 and Fig 3). Finally, experimental signals in which we modified the energy spectrum by randomly changing the amplitude level of each harmonic (*sort*) did not elicit strong responses as the synthetic control signals (p < 0.002, see Table 1 and Fig 3). Indeed, 11 out of 15 tested pups did not respond to these signals (no call produced, no look).

### Propagation

#### Analyses of propagated signal

For each of the 3 studied parameters, we found that measurements at 1m and 8m were strongly correlated (r coefficients of Pearson greater than 0.72; Table 2), meaning that signal structure was not significantly modified. However, when distance increased, these correlations became weaker, close to zero for both averaged energy spectra and envelopes. On the contrary, the frequency modulation pattern from signals recorded at 1 m and those from propagated signals still showed strong correlations up to 64m (i.e., r between 0.88 and 0.67 –Table 2). At 128m, it was impossible to measure the frequency modulation from the recordings, the signal being below the background noise.

**Table 2:** Correlations of spectra, envelopes and FM between signals propagated at 1 m (control) and those broadcast at 8, 32, 64 and 128 meters. Only frequency modulations show strong correlations to the control at long range (64m), showing that FM pattern is highly resistant to degradation during propagation. (-: non measurable). Correlations in bold show p- values < 0.05.

Correlations/ 1m	8m	32m	64m	128m
Averaged spectra (n = 12)	**0.78**	0.53	0.26	0.05
Averaged envelopes (n = 12)	**0.72**	0.34	0.48	0.09
Average FM (n = 12)	**0.88**	**0.76**	**0.67**	-

#### Playback Tests at Different Distances

**Fig 4:**
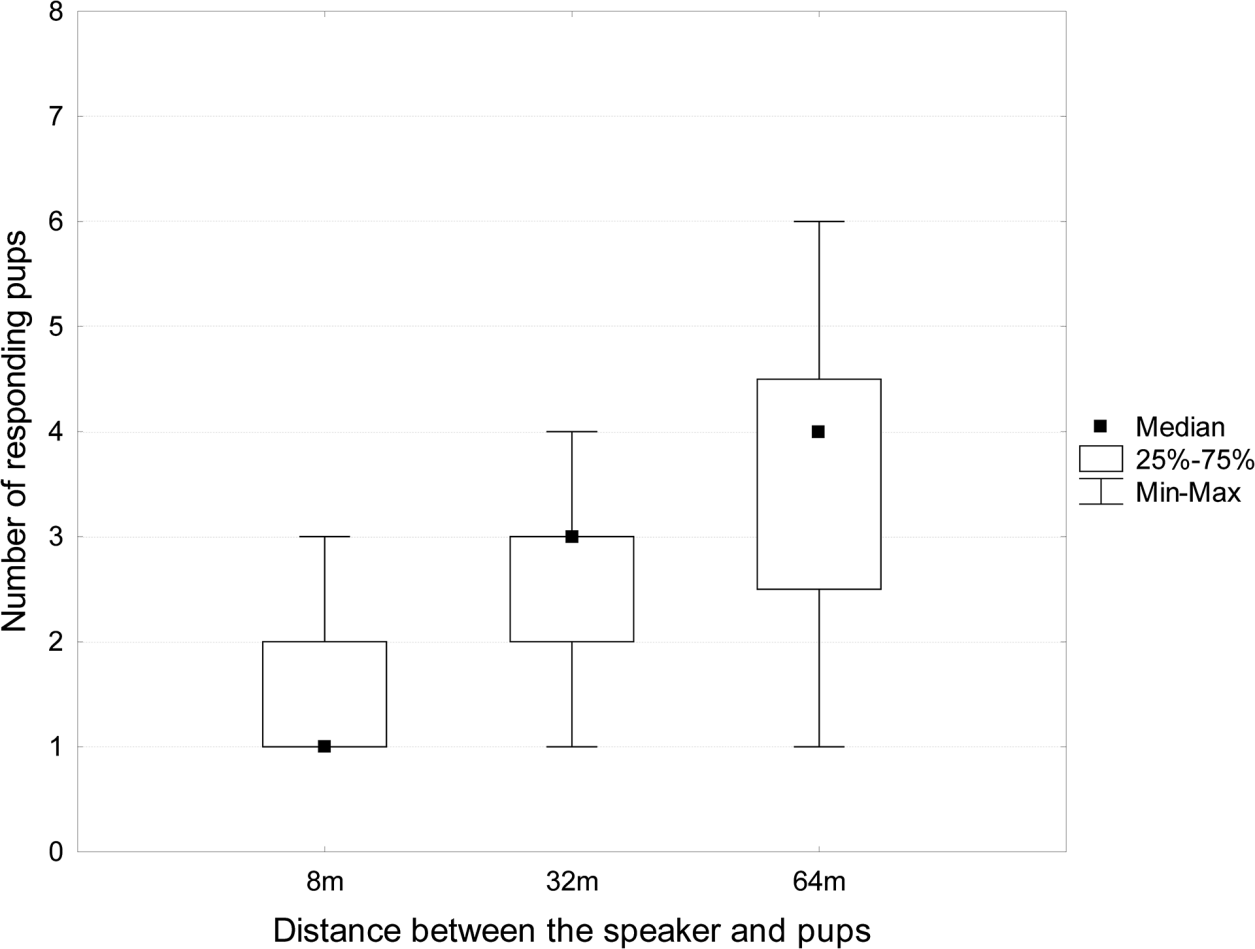
Number of pups within a group responding to female calls at 3 different distances. When the distance decreases, the number of responding pups decreased too. For all tested distances, the filial pup of the female chosen for the playback always responded.

Playback tests performed on different groups of pups at different distances (8, 32 and 64m, Fig 4) revealed that the number of responding pups significantly decreased with shorter distances (Friedman ANOVA (n = 12, df = 2) = 11.91, p = 0.0026). At 64 meters, 3.6 pups on average (± 1.6, SE) responded to a given female’s calls, at 8 meters only 1.5 pups responded (± 0.65, SE), and among them, there was always the pup of the female from which the calls came. Moreover, there were significant differences between the number of responding pups at 8 and those at 32m and 64m (Wilcoxon matched pair tests, p = 0.033 and p = 0.0067 respectively), this was not the case between tests at 32 and 64m (Wilcoxon matched pair test, p = 0.058 respectively).

## Discussion

### Individual Vocal Signature

Playback experiments with synthetic signals clearly show that several acoustic parameters are used by Antarctic fur seal (AFS) pups to identify their mother’s voice. Both amplitude and frequency modulations are essential since all experimental signals in which one of these parameters was removed (*wam or wfm*) or modified (*rev*) were not recognised by pups as calls produced by their mother. The use of FM in individual recognition has been demonstrated in several colonial species (in penguins: [28]; in gulls: [29]; in pinnipeds: [12,17,16,30]). This parameter is particularly well adapted to colonial environment as it is highly resistant to degradation during propagation [31]. We also found that amplitude modulations are essential in individual recognition. Most female calls contain both slow and fast amplitude modulations that can be used for individual recognition, but probably only at short distances as AM are strongly degraded during propagation [32,33]. However, AM can be used by the pup to localise its mother in the colony [34] and estimate distances [35]. This is important because even though Antarctic fur seal mothers and pups tend to return to the birth site, this suckling site can be moved by more than 10m [23]. Moreover, when getting older, pups became more mobile in the colony and thus they can be far from their birth site.

Another crucial parameter highlighted by the playback experiments is the energy spectrum. Indeed, experimental signals in which the relative level of harmonics has been changed (*wam* or *sort*) were not recognised by pups, suggesting that pups analysed the relative amplitude levels of harmonics to identify their mother’s voice. However, pups did not use the exact frequency values of harmonics or the frequency distance between harmonics. Indeed, individual recognition was only impaired with highly shifted signals (a300 or l150-l300). This highlights that pups use the spectral profile of their mother’s calls, but as Subantarctic fur seal pups [17], they do not perform a precise frequency analysis of their mother’s calls. In summary, pups performed an accurate temporal analysis (AM and FM) but also a precise spectral analysis. The use of a multi-parametric individual vocal signature is particularly well adapted in colonial environments. Indeed previous studies on colonial birds and gregarious mammals have shown that animals use multiple acoustic features to individually identify each other when they are communicating in such environments [17,28,29,16,30,36]. If a given parameter is not reliable (e.g., at long distances or with a high background noise), the use of alternative parameters could ensure individual identification.

### A Two-Step Individual Recognition Process

To test the efficiency of the individual vocal signature, we performed two types of tests. One consisted of measuring the degradations of the 3 main parameters composing the individual signature (AM, FM and energy spectrum) at different distances of propagation, and, the second one was aiming to test the responsiveness of pups to these same propagation distances. The first experiment demonstrated that AM and energy spectrum were strongly degraded beyond 8m (coefficients of correlations lesser than 0.50 at 32m), whereas FM were highly resistant to these degradations up to 64m. This is not surprising since we know that AM and high-pitched frequencies are strongly degraded when propagated at long distances whereas slow FM are very resistant to these effects [31]. This suggests that the individual recognition process is a two-step mechanism: at long range, pups can only rely on the FM pattern to identify their mother’s voice, and when they get closer to the source, they can use other additional features such as the energy spectrum and/or AM to finalize the identification process. This hypothesis seems to be supported by our second experiment. Indeed, at long distances, several pups (systematically including the pup whose mother calls were broadcast) are responding to a given female’s calls whereas at short distances (8m), 1 or 2 pups only are responding to the same calls, one being the filial pup of the mother. Considering the results of our propagation experiments, at long range (32 and 64m) both spectral and AM features were degraded and thus the degradation of mother calls seems to induce some errors of recognition in pups even if one parameter, the FM pattern, is still reliable. One single parameter may thus not be enough to allow a highly reliable identification at long range and in a noisy environment. The redundancy of information makes the individual identification more reliable but only for medium or close distances. Further experiments on single pups using mother and non-mother calls are needed to fully support this result.

The interactions between the propagation properties of acoustic features and the environmental constraints have to be taken into account to better understand the mechanisms and adaptations at the level of information coding. If propagation experiments have been performed in other species, this is the first time that such two-step process has been experimentally demonstrated in a mammal species.

### Comparison with Other Pinniped Studies

The comparison with other pinnipeds species is limited, since individual vocal signatures have only been investigated in two other pinniped species: the Subantarctic fur seal (SFS), *Arctocephalus tropicalis*, and the Australian sea lion (ASL), *Neophoca cinerea*. SFS pups used both FM and energy spectrum to identify their mother’s voice [17], whereas AM and frequencies values were not involved in the identification process. In ASL, pups used AM, FM and the exact frequency values of the calls, but not the energy spectrum [36].

From these three species, we can highlight the common use of frequency modulations in their vocal signature, which can be easily explained by the fact that these species encounter the same environmental constraints: they live in colony where both the risk of confusion and the background noise are high. They also show the same social constraints: their mothers alternate foraging trips at sea with ashore periods, so the need for an accurate mother’s voice recognition by pups is primordial in all species.

The differences found between vocal signature components could potentially be explained by differences in call structure and natural habitats. For instance, both AFS and ASL females’ calls show fast and slow AM which is not the case in SFS (no fast AM, Charrier *pers*. *obs*). Fast AM may be of a great help for the pups to locate their mothers in the colony in both these species. Indeed, the habitat in which AFS breeds in Kerguelen Archipelago is an open-area composed of sandy beach and meadows, those of *ASL* on Kangaroo Island is also an open-area mainly composed of sandy beach and loose bushes [36], whereas those occupied by SFS on Amsterdam Island is a rocky beach. The use of AM in such obstructed area is not reliable. The use of exact frequency values in ASL could be explained by a different degree of colonial density. Indeed, even if colonial, most ASL colonies show a low density of animals compared to any fur seal colony. The confusion between individuals is thus much lower, and thus a frequency analysis of the calls can be sufficient for pups to identify their mothers among a limited number of females, as it has been shown in some colonial birds (nesting penguins: [28]; lariids: [29]).

From these studies, we can suggest that individual recognition system in colonial mammals is strongly correlated to environmental constraints, as it has been shown in colonial seabirds [28]. Further studies with mammal species showing weak and extreme ecological constraints are essential to confirm such relationship, and to better understand the evolution of communication in vertebrates.

## Supporting Information

S1 Sound Files Examples of natural and experimental female signals played-back to pups. f11nat and f11synt: natural and synthetic control signals of female f11. f9synt: synthetic control of female f9; f9wam: synthetic control signal without amplitude modulation; f9wfm: synthetic control signal without frequency modulation; f9sort: synthetic control signal with amplitude level of each harmonic randomly changed; f9l75 & f9l300: synthetic control signal linearly shifted upwards by 75 Hz and 300 Hz respectively; f9a75: synthetic control signal with fundamental frequency shifted upwards by 75 Hz and harmonic series rebuilt.
(ZIP)
